# Attitudes towards Mandatory Occupational Vaccination and Intention to Get COVID-19 Vaccine during the First Pandemic Wave among Mongolian Healthcare Workers: A Cross-Sectional Survey

**DOI:** 10.3390/ijerph19010329

**Published:** 2021-12-29

**Authors:** Battsetseg Turbat, Bold Sharavyn, Feng-Jen Tsai

**Affiliations:** 1 Ph.D. Program in Global Health and Health Security, College of Public Health, Taipei Medical University, Taipei 11031, Taiwan; jijee.promise20@gmail.com; 2 Department of Nursing, Mongolian University of Pharmaceutical Sciences, Ulaanbaatar 18130, Mongolia; 3 Department of Traditional Medicine, International School of Mongolian Medicine, Mongolian National University of Medical Sciences, Ulaanbaatar 14210, Mongolia; boldshrv@gmail.com; 4 Master’s Program in Global Health and Development, College of Public Health, Taipei Medical University, Taipei 11031, Taiwan

**Keywords:** healthcare workers, occupational vaccination, COVID-19, vaccine, attitude towards a vaccine

## Abstract

Mandatory occupational vaccination for health care workers (HCWs) is a debatable issue, especially during the COVID-19 pandemic. This study aimed to determine Mongolian HCWs’ attitudes towards mandatory occupational vaccination, the intention to get the COVID-19 vaccine, and the associated factors. A cross-sectional study based on an online survey with a convenience sampling strategy was conducted from February to April 2021 among 238 Mongolia HCWs. Chi-square and logistic regression were performed for analysis. While only 39.9% of HCWs were aware of recommended occupational vaccinations, they highly agreed with the mandatory occupational vaccination on HCWs (93.7%). The agreement rate is significantly higher than their attitude toward general vaccination (93.7% vs. 77.8%). HCW’s willingness to get the COVID-19 vaccine was high (67.2%). HCWs aged 26–35 years old who worked in tertiary level hospitals had less willingness to get the COVID-19 vaccine (50%). Participants with lower confidence in the efficacy of the COVID-19 vaccine (ORs = 15.659) and less positive attitudes toward general vaccination (ORs = 5.288) were less likely to get the COVID-19 vaccine. Mongolian HCWs’ agreement rate of mandatory occupational vaccination is higher than other countries. Their intention to get the COVID-19 vaccine is high and associated with confidence in the effectiveness of the vaccine.

## 1. Introduction

Healthcare workers (HCWs) are the main force in the health services sector. Except for general occupational hazards, HCWs are specially facing biological hazards such as infectious disease viruses in their work environment. Like other workers, the right to health of HCWs is regulated and protected by the Promotional Framework for Occupational Safety and Health Convention established by the International Labor Office (ILO). For preventing HCWs’ special occupational hazards, the World Health Organization (WHO) not only provides the vaccine recommendation but also collaborated with ILO to provide a framework and the Occupational Health Program to Health Workers [1,2,3]. However, the national vaccination policy for HCWs varied among countries and the vaccine coverage rates among HCWs are also diverse. 

Prior research has thoroughly investigated immunization coverage of recommended vaccines for HCWs and found significant immunity gaps occur among HCWs, especially for measles and influenza [4,5,6,7,8,9,10,11,12,13]. To reduce the immunity gap, there is an ongoing debate on mandatory vaccination approaches for HCWs in European countries, the USA, and others [5,14,15,16,17,18,19,20,21]. The ethical dilemma represented by the mandatory vaccination on HCWs is the balance between HCWs’ autonomy and patients’ welfare, public health, and HCWs’ own health interests [14,22,23,24,25]. Broadly, most agree that HCWs should behave in their patients’ best interest, which includes staying healthy in order to continue caring for them, but this should not limit HCWs’ individual rights [24]. Besides, the patients who insist on being cared for by HCWs who are vaccinated against vaccine-preventable infections must acknowledge that their autonomy is equal to that of their healthcare providers [25]. While mandatory immunization may be controversial, the fact remains that opt-in, voluntary programs appear to be more successful and acceptable among most involved parties [15,16,17]. Regarding HCWs’ attitudes towards mandatory vaccination policy, the support of such an approach highly depended on the type of disease [22,23,26,27,28]. However, those studies are conducted only in Western countries with developed status of the health care system. The voice of HCWs regarding the issue in developing countries was lacking from literature even though the previous review found that HCWs in low-middle-income countries (LMICs) were widely exposed to occupational hazards without sufficient protection. 

The emerging COVID-19 pandemic reactivated the discussion of mandatory vaccination on HCWs due to the fact that they were at high risk for COVID-19 infection [29,30,31]. There were already 2,679,563 COVID-19 cases and 7857 deaths reported among HCWs [32]. With that risk, HCWs are among the priority to receive the vaccine around the world [30,33,34]. Their attitudes toward COVID-19 vaccination were not only important in allowing them to treat COVID-19 patients, but also in shaping the public’s attitudes toward vaccination [33,35,36]. However, previous studies regarding COVID-19 vaccine acceptance among healthcare workers showed the acceptance rate ranged from 27.7% to 78% in different countries [36,37] and such acceptance rates are comparatively lower than general populations reported globally which reflected the vaccine hesitancy among healthcare workers [37]. Responding to the immunization gap, Italian healthcare workers were the first in Europe to get mandated COVID-19 vaccination [38]. In Australia, Bradfield and Giubilini (2021) suggested a conditional COVID-19 vaccination policy for healthcare workers who refuse to receive the vaccine [39]. However, such discussion is not yet heard from developing countries, especially from Asia. 

As a recognized model in LMIC countries, Mongolia had no local COVID-19 cases before November 2020 [40]. However, after the first domestic case reported in Mongolia on 11 November 2020, 349,866 COVID-19 cases have been reported with 1694 deaths under high alert levels and several lockdowns. [32,41,42] Moreover, by November 2021, around 4138 cases of COVID-19 were reported among HCWs. [32] With purchased vaccines from COVAX and received vaccines from China and Russia, the Mongolian government aimed to vaccinate 60 percent of its population towards ending the pandemic. [43,44,45] As in other countries, the health workforce is the first target group for getting the COVID-19 vaccine in Mongolia and the vaccine campaign started on 23 February 2021. [43,44] Though the vaccine coverage rate for the traditionally recommended vaccine was high in Mongolia, there is a knowledge gap regarding Mongolian HCWs’ attitudes toward mandatory vaccination on themselves, especially for a newly developed vaccine such as the COVID-19 vaccine. Therefore, we conducted the study to evaluate attitudes towards mandatory occupational vaccination and intentions to get the COVID-19 vaccine among Mongolian HCWs. 

## 2. Materials and Methods

### 2.1. Research Design

The cross-sectional anonymous online survey assessing HCWs’ knowledge of recommended occupational vaccination for HCWs, attitudes toward general vaccination and approach of vaccination in practice, and risk perceptions and intentions to get the COVID-19 vaccine was conducted from 18 February to 23 April 2021. Participants were healthcare workers aged above 18 years old and currently working in public hospitals. 

We first uploaded the questionnaire into SurveyCake software, an online survey tool widely used in Taiwan. [46] Then, a convenience sampling strategy was applied. In detail, we shared the survey invitations and link generated by SurveyCake with contacted individuals working in government primary care hospitals, provincial and district hospitals, and referral and specialized hospitals via their email and individual messaging programs (Facebook messenger) to target participants. IP address restriction technology was used to ensure that users with the same IP address could only complete the questionnaire once. A total of 1576 HCWs viewed the questionnaire, and 238 respondents completed the survey. The effective response rate was 15.0%. All participants were included in the final analysis. 

The study was approved by the Ethics Committee on the IRB Committee of Taipei Medical University (No: N202011051) and Mongolian University of Pharmaceutical Sciences (No:1). 

### 2.2. Measures

This self-administered online questionnaire consists of three parts.

#### 2.2.1. Individual Characteristic

Participants’ information of age, gender, educational level, place of work, occupation, work experience, and the monthly salary was collected in the first part. While their working hospital level might matter in their exposure risk, we also collected the information of their working hospital level for analysis. The Mongolian public hospital system consists of health facilities offering different levels of service: (1) primary care level, including family health center, soum health center, inter-soum hospital; (2) secondary level, including Aimag general hospital, district general hospital, maternity hospital, ambulance care center; and (3) tertiary level, including regional diagnostic and treatment centers, central hospital, and single specialty center [47].

#### 2.2.2. Knowledge of Recommended Occupational Vaccinations and Attitude toward General Vaccination and Approach to Vaccination in Practice

The second part contained questions regarding “knowledge of recommended occupational vaccinations”, their attitudes toward “general vaccination” and “approach to vaccination in practice”. [9] The question on knowledge of occupational vaccination referred to participants’ understanding of recommended vaccinations for health care workers by WHO [48] and by the Ministry of Health Mongolia (MOH) in 2019. [49,50] The WHO recommended 10 vaccinations for HCWs which are measles, polio, rubella, pertussis, influenza, BCG, Hepatitis B, varicella, diphtheria, and meningococcal vaccine. [48] Following the immunization policy recommended by WHO, there are 7 recommended occupational vaccinations for HCWs in Mongolia: hepatitis B, tuberculosis, polio, tetanus, diphtheria, pertussis, and influenza. [49,50,51] Participants were asked if the vaccine was recommended for healthcare workers by WHO and by the MOH. Yes/no/do not know answers were provided for a response for each vaccine. Participants’ answers were summed up to be their knowledge score regarding recommended occupational vaccination by WHO and by the MOH (1 point for each correct answer, 0 for incorrect answers including no and do not know). The scores were further divided into “well” and “low” knowledge groups by cut-off point as mean score for analysis. 

Regarding participants’ attitudes toward general vaccination, they were asked the question “Please self-rate your attitude towards vaccinations in general”. A five-point Likert scale ranging from 1 “strongly against” to 5 “strongly favorable” was provided for response. There were 77.8% participants who chose “favorable” and “strongly favorable” on the scale. However, the scale was further divided into the “more favorable/positive” and “less favorable/positive” groups by cut-off point as the median for further analysis. For their attitudes toward the approach to vaccination in practice, they were asked “Do you agree with the approach of requiring HCWs to get vaccination due to their work?” and “Do you agree that mandatory approach of vaccination for HCWs is needed?”. In answering those questions, participants were asked to focus on vaccines in general rather than specific vaccines. Yes/no answers were provided for response.

#### 2.2.3. Risk Perception and Intention to Get COVID-19 Vaccine

The final section of the questionnaire contained scales measuring participants’ risk perceptions which included “perceived severity”, “susceptibility of COVID-19 disease”, “perceived effectiveness of COVID-19 vaccine”, and their “intention to get COVID-19 vaccine”. The original questionnaire developed by Ling et al. (2019) was used in a previous study for flu vaccine with sufficient internal consistency. [52] Because influenza and COVID-19 are diseases with similar transmission routes and the vaccines are both newly developed without sufficient vaccination history, [53] we modified the questionnaire to evaluate the risk perception and intention of COVID-19 vaccination in our study. While information regarding the COVID-19 vaccine policy in Mongolia was not fully determined during the development of the questionnaire, we only picked a questionnaire regarding risk perception and vaccination intention for this study. In the current study. The Cronbach’s alpha was 0.81 in our study.

The participants’ perceived severity of COVID-19 disease subscale contains three items designed to measure participants’ agreement regarding the three statements as (1) “COVID-19 can be a life-threatening illness”, (2) “The negative impact of COVID-19 is very severe”, and (3) “COVID-19 is a serious illness for someone like me.” Responses were based on a 5-point Likert scale ranging from 5 (strongly agree) to 1 (strongly disagree). The three items were summed up to form a composite score and divided into “low” and “high” severity of COVID-19 disease groups by cut-off point as mean score. 

Participants’ perceived susceptibility to COVID-19 disease was evaluated by the question: “Without a COVID-19 vaccine, I will be vulnerable to contract COVID-19 in the next COVID-19 wave”. Responses were based on a 5-point Likert scale ranging from 5 “strongly agree” to 1 “strongly disagree”. The scale was divided into “low” and “high” susceptibility to COVID-19 disease groups by cut-off point as the mean score for analysis.

Participants’ perceived efficacy of the COVID-19 vaccine subscale contains three items intended to measure the participant’s agreement regarding the three statements as (1) “Vaccination is a very effective way to protect me against the COVID-19”, (2) “If I don’t get a COVID-19 vaccination I will be at risk of catching COVID-19 in the next COVID-19 wave”, and (3) “I’m sure that having a COVID-19 vaccine would be effective in reducing my personal risk of contracting COVID-19”. Responses were based on a 5-point Likert scale, ranging from 5 “strongly agree” to 1” strongly disagree”. The three items were summed up to create a composite score and further divided into “low” and “high” groups by cut-off point as the mean score for analysis. 

Participants’ intentions to get the COVID-19 vaccine were evaluated by the question: “I intend to have a COVID-19 vaccination”. Responses were based on a 5-point Likert scale ranging from 5 “strongly agree” to 1 “strongly disagree”. To analyze the contributing factors to the intention, we divided the score into “less” and “more” intention groups by the mean score. 

### 2.3. Statistical Analysis

Chi-square test was used to compare individual characteristics, including age, gender, educational level, place of work, occupation, work experience, monthly salary, and intention to vaccinate against COVID-19, as well as “knowledge of recommended occupational vaccinations”, their attitudes toward “general vaccination” and “approach to vaccination in practice”, their “perceived severity of the COVID-19 disease”, “perceived susceptibility of COVID-19 disease“, and “perceived efficacy of COVID-19 vaccine” between participants’ intentions to get COVID-19 vaccine groups.

Bivariate analysis was initially used to understand the relationship between participants’ intentions to get COVID-19 vaccine and potential confounding factors including age, gender, educational level, place of work, occupation, work experience, monthly salary, knowledge of recommended occupational vaccinations, attitudes toward general vaccination, and approach to vaccination in practice. Then logistic regression was applied for categorical variables to determine the statistical significance of the association between participants’ risk perceptions and intentions to get vaccinated against COVID-19 by controlling the significantly impacted confounder found from bivariate analysis. 

Odds ratios (ORs) and 95% confidence intervals (CIs) were calculated, and the significance level was set at 0.05. All statistical analyses were performed in SPSS version 23.

## 3. Results

### 3.1. Comparison of Demographic Characteristics between Intention to Get COVID-19 Vaccine Group

Table 1 shows the demographic data of the participants. The majority of participants were women (181.9%) aged between 26–35 years (62.2%) with educational level as bachelor’s degree (56.7%). Moreover, most of them worked as physicians (68.1%) in tertiary-level hospitals (46.6%). With work experience around 0–5 years (37.8%), their monthly salary ranged between MNT 245.60–385.92 (60.9%). From the original response of intention of getting the COVID-19 vaccine reported by participants, 87.9% of participants answered, “strongly agree” and “agree”, and the mean score of our HCWs for the intention is high at 4.46 which reflects a high intention of getting the COVID-19 vaccine among Mongolian HCWs. When grouping them for analysis, 67.2% of participants who answered, “strongly agree” to the question were classified in the “more intention” group while the others were classified in the “less intention group”. 

For comparison between different intentions to get COVID-19 vaccine groups, occupational status was the only factor that was significantly different between groups. The percentage of participants with occupations other than physicians was significantly higher among those with less intention to get vaccinated against COVID-19. Age, gender, education, workplace, job experience, and the monthly salary of healthcare workers were not significantly different between less and more intention groups. 

### 3.2. Comparison of Knowledge of Recommended Occupational Vaccinations, Attitudes toward General Vaccination, and Approach to Vaccination in Practice between COVID-19 Vaccine Intention Groups

In Table 2, we present the comparison results of knowledge of recommended occupational vaccinations, attitude toward general vaccination, and approach to vaccination in practice between COVID-19 vaccine intention groups. Overall, Mongolian healthcare workers’ knowledge of recommended occupational vaccinations was insufficient in that less than half of the participants answered correctly for recommended occupational vaccinations by WHO (2019) and by the Minister of Health Mongolia. In terms of knowledge of recommended vaccinations for HCWs by WHO (2019), the top 2 known occupational vaccinations for HCWs were influenza vaccine (90.3%) and viral hepatitis B vaccine (88.7%), followed by measles vaccine (47.5%) and polio (19.7%). Among the recommended vaccines by the MOH, the top 2 known occupational vaccines for HCWs were also influenza vaccine (90.3%) and viral hepatitis B vaccine (88.7%), followed by BCG vaccine (38.7%). All recommended occupational vaccinations and the responses regarding the knowledge of the participants are listed in the Supplementary Materials. 

Regarding participants’ attitudes toward a general vaccine, 43.3% of participants hold a more positive attitude while 56.74% had a less positive attitude towards general vaccination. Regarding participants’ attitudes toward the approach to vaccination in practice, more than 95% of the participants agreed with the vaccination policy that HCWs must be vaccinated due to their work and about 93.7% of them agreed with the mandatory vaccination policy for HCWs. 

Between groups, the percentage of participants with low knowledge about the recommended vaccinations of WHO (2019) and MOH is higher in the group with more intention to get vaccinated against COVID-19. In addition, participants’ attitudes toward general vaccination and approach to vaccination in practice differed significantly between both COVID-19 intention groups. The percentage of participants with less positive attitudes toward general vaccination was significantly higher in the group with less intention to vaccinate against COVID-19 (83.3%). But, reversed results were shown for the percentage of participants agreeing with the statement of requiring HCWs to get vaccination due to their work (98.8%) and the statement of mandatory vaccination approach among HCWs (96.3%). 

### 3.3. Comparison of Risk Perceptions between COVID-19 Vaccine Intention Groups

Table 3 shows the comparison of risk perception between COVID-19 vaccination intention groups. Over 62.2% of participants expressed strong concerns about the high severity of COVID-19 disease and 64.9% of participants expressed strong concerns about the susceptibility of COVID-19 disease. In addition, 57.6% of participants expressed high confidence in the effectiveness of the COVID-19 vaccine, while 42.4% expressed low confidence in the effectiveness of the COVID-19 vaccine. Perceived severity and susceptibility to COVID-19 disease and perceived efficacy of COVID-19 vaccine differed significantly between COVID-19 vaccination intention groups. The percentage of participants with low concern about severity and susceptibility to COVID-19 disease and low confidence in the efficacy of the COVID-19 vaccine was significantly higher in the group with less intention to get COVID-19 vaccine.

### 3.4. Associations between Demographic Characteristics, Attitude toward General Vaccination, Risk Perceptions, and COVID-19 Vaccination Intention

In Table 4, we present the relationship between participants’ intention to be vaccinated against COVID-19 and their demographic characteristics, attitude toward general vaccination, and risk perception. Participants’ attitudes toward general vaccination and the perceived effectiveness of the COVID-19 vaccine were significantly associated with their intention to be vaccinated against COVID-19. Participants who had a less positive attitude toward general vaccination had significantly higher ORs (OR = 5.288, 95% CI 2.266–12.339) for less intention to get vaccinated against COVID-19. Participants who had lower confidence in the efficacy of the COVID-19 vaccine had significantly higher ORs (OR = 15.659, 95% CI 6.798–36.070) for less intention to get vaccinated against COVID-19.

## 4. Discussion

To the best of our knowledge, our study is the first to focus on the issue of immunization for healthcare workers in Mongolia. This study found that Mongolian healthcare workers highly agreed with the mandatory vaccination policy for HCWs in general. The agreement rate is significantly higher than their attitude toward general vaccination (93.7% vs. 77.8%). In addition, the agreement rate is also higher than the findings from Germany (68.4%) and Greece (63–70.6%). [7,22,27] While the mandatory vaccination approach of healthcare workers is still a disputable issue around the world [4,5,14,15,16,18,19], this result provides a clue for Mongolia’s vaccination policy in enhancing the vaccine coverage rate among HCWs. Since Mongolia successfully transitioned from a communist state to democracy in the 1990s, previous research found that the majority of Mongolians are still rooted in authoritarianism. [54] As a result, it might make them more likely to comply with authority and act accordingly. Further study is needed to understand the reason behind this phenomenon and HCWs’ opinions on the vaccines of other diseases. 

From the result, the HCWs’ agreement rate of mandatory vaccination is significantly higher than their attitudes toward general vaccination (93.7% vs. 77.8%) which reflects a COVID-19 vaccine attitude—intention gap of 15.9%. The possible explanation for this gap might be that healthcare workers consider mandatory vaccination policy as part of the job responsibilities of their profession [55]; however, they answered the question regarding intention to get the vaccine from their individual perspective. While HCWs’ rights as individuals should be respected and their autonomy is equal to patients, our result reflected the need to consider their concerns as individuals [24,25].

In addition, we found poor knowledge of recommended occupational vaccination by WHO and by the MOH among HCWs in Mongolia, except influenza and hepatitis B vaccination. Similar to earlier studies conducted in France and Italy, we found a high recall rate for recommended occupational vaccination for hepatitis B and influenza [9,56,57]. The HCW hepatitis B vaccination campaign under the HPCE program (National Hepatitis Prevention, Control, and Elimination program) and the new act regulating the guidelines for vaccinating HCWs to prevent hepatitis B in 2018 might be the reason for the phenomenon. [49,58] Similarly, HCWs correctly identified influenza vaccination due to the fact that influenza vaccination was recommended for HCWs each year and a big campaign was organized in 2020. [51] Future research on determining the real coverage and acceptance of Influenza vaccination among HCWs is recommended. Moreover, the poor knowledge of occupational vaccination for HCWs would require detailed national recommendations for vaccination of healthcare workers in Mongolia.

Our survey showed that younger HCWs (66.7%) and those who work in tertiary care hospitals (50.0%) were less likely to get a vaccination against COVID-19. Young people’s general mindset that they are healthy with good immune systems might be the reason for the phenomenon. This is similar to previous studies in which it was shown that older HCWs would be more likely to get the COVID-19 vaccine due to their risk of infection and severity of the infection [35,36]. In addition, while the number of daily confirmed COVID-19 cases was low and the disease was treated in a few specialized hospitals in Mongolia, HCWs working in tertiary care hospitals were less likely to get vaccinated. 

Interestingly, participants who agreed more with the need to vaccinate HCWs due to their occupational risk have more intention to take the COVID-19 vaccine. Moreover, our findings showed a comparatively high COVID-19 vaccination intention among Mongolian healthcare workers in that 87.8% of them expressed intention to get the COVID-19 vaccine. The vaccine hesitancy of Mongolian healthcare workers is lower when compared with HCWs’ vaccine hesitancy reported in Asian countries and other regions. [59,60,61] This phenomenon may be explained by three reasons: (1) the new confirmed COVID-19 cases in Mongolia started to rise rapidly at that time which might have led to high worries of getting COVID-19 among healthcare workers (risk of infection); (2) by February 2021, there were 185 HCWs infected with COVID-19, counted as 6.3% of all new confirmed cases. Additionally, Mongolian healthcare workers had inadequate personal protective equipment, including an N95 mask and face shield. [42] This fact might have led to the increase in perceived risk to get COVID-19 infection among HCWs. Therefore, they showed more intention to get the COVID-19 vaccine to protect themselves. 

Regarding COVID-19 vaccination intention and the related factors, our findings showed a similar result to previous studies in that HCWs with a more positive attitude toward vaccines had a greater intention to get vaccinated. [62] This finding is echoed in the risk perception [63] and protection motivation theory. [64] Similar to previous studies that hazard-specific risk perceptions predict vaccination behavior, [65] we found participants who had higher confidence in the effectiveness of the COVID-19 vaccine were more likely to receive the vaccine. [59,61,66] Surprisingly, participants’ perceived severity and susceptibility were not significantly associated with their intention to get the COVID-19 vaccine after adjusting for other factors. The overlapping of participants with high perceived severity and susceptibility and high confidence in the COVID-19 vaccine might be the reason for the phenomenon. 

### Limitations

This study had several limitations. Firstly, the low response rate during epidemics in this research might lead to possible selection bias. Secondly, our investigation period was from February to April 2021, and it overlapped with the local COVID-19 outbreak peak. Therefore, our study result might overestimate or underestimate HCWs’ acceptance of the COVID-19 vaccine. Third, in this study, we excluded healthcare workers who work in private hospitals. Finally, with the nature of cross-sectional design, the study result could be seen as associations rather than causal relationships. Furthermore, we cannot rule out the reporting bias due to the study design as a self-reported survey.

## 5. Conclusions

Our findings indicate that Mongolian HCWs’ agreement rate with the mandatory policy of vaccination on HCWs was surprisingly high, even higher than their attitudes toward general vaccination. Moreover, their COVID-19 vaccine acceptance was high. The participants with a less positive attitude toward general vaccines and less confidence in the effectiveness of the COVID-19 vaccine had less intention to get the vaccine. A follow-up study regarding vaccination coverage rates among HCWs is recommended to see if there is a gap in the intention of vaccination and real action.

## Figures and Tables

**Table 1:** Demographic characteristics of intention to be vaccinated against COVID-19.

Characteristic	Total		Less Intention		More Intention		*p*-Value
	(*n* = 238)		(*n* = 78)		(*n* = 160)		
	*n*	%	*n*	%	*n*	%	
**Age groups**							
18–25	18	7.6	8	10.3	10	6.3	0.31
26–35	148	62.2	52	66.7	96	60.0	
36–45	48	20.2	14	17.9	34	21.3	
46–55	20	8.4	3	3.8	17	10.6	
Over 55	4	1.7	1	1.3	3	1.9	
**Gender**							
Women	195	81.9	64	82.1	131	81.9	0.97
Men	43	18.1	14	17.9	29	18.1	
**Education**							
Less than bachelor	13	5.5	6	7.7	7	4.4	0.47
Bachelor	135	56.7	41	52.6	94	58.8	
Above Bachelor	90	37.8	31	39.7	59	36.9	
**Worksite**							
Primary health care level	32	13.4	6	7.7	26	16.3	0.19
Secondary level	95	39.9	33	42.3	62	38.8	
Tertiary level	111	46.6	39	50.0	72	45.0	
**Occupation**							
Physician	162	68.1	45	57.7	117	73.1	0.02 *
Other	76	31.9	33	42.3	43	26.9	
**Job experience**							
0–5 years	90	37.8	31	39.7	59	36.9	0.50
6–10 years	70	29.4	24	30.8	46	28.7	
11–20 years	56	23.5	19	24.4	37	23.1	
over 20 years	22	9.2	4	5.1	18	11.3	
**Monthly salary**							
Lower than USD 245.60	44	18.5	13	16.7	31	19.4	0.61
USD 245.60–385.92	145	60.9	51	65.4	94	58.8	
Over than USD 385.92	49	20.6	14	17.9	35	21.9	

Significant level: * *p* < 0.05.

**Table 2:** Comparison of knowledge about the recommended occupational vaccine, practical vaccination behaviors, and attitudes toward the general vaccine among COVID-19 vaccination intention groups.

Variables	Total		Less Intention		More Intention		*p*-Value
	(*n* = 238)		(*n* = 78)		(*n* = 160)		
	*n*	%	*n*	%	*n*	%	
**Knowledge**							
10 recommended vaccinations for HCWs by WHO (2019)							
Low	143	60.1	45	57.7	98	61.3	0.60
Well	95	39.9	33	42.3	62	38.8	
MOH recommended vaccination plus flu for HCWs in Mongolia							
Low	139	58.4	45	57.7	94	58.8	0.88
Well	99	41.6	33	42.3	66	41.3	
**Attitudes toward general vaccination**							
Less positive	135	56.7	65	83.3	70	43.8	0.000 ***
More positive	103	43.3	13	16.7	90	56.3	
**Approach to vaccination in practice**							
Agree with the approach of requiring HCWs to get vaccination due to their work							
No	10	4.2	8	10.3	2	1.3	0.001 **
Yes	228	95.8	70	89.7	158	98.8	
Agree that a mandatory approach to vaccination for HCWs is needed							
No	15	6.3	9	11.5	6	3.8	0.02 *
Yes	223	93.7	69	88.5	154	96.3	

Significance level: * *p* < 0.05, ** *p* < 0.01, *** *p* < 0.001. MOH: Ministry of Health; HCWs: Health care workers; WHO: World Health Organization.

**Table 3:** Comparison of risk perception and perceived effectiveness between groups intending to receive COVID-19 vaccination.

Risk Perceptions	Total		Less Intention		More Intention		*p*-Value
	(*n* = 238)		(*n* = 78)		(*n* = 160)		
	*n*	%	*n*	%	*n*	%	
Perceived severity of COVID-19 disease							
Low	90	37.8	41	52.6	49	30.6	0.001 ***
High	148	62.2	37	47.4	111	69.4	
Perceived susceptibility of COVID-19 disease							
Low	84	35.3	39	50.0	45	28.1	0.001 ***
High	154	64.7	39	50.0	115	71.9	
Perceived efficacy of COVID-19 vaccine							
Low	101	42.4	65	83.3	36	22.5	0.000 ***
High	137	57.6	13	16.7	124	77.5	

Significant level: *** *p* < 0.001.

**Table 4:** Associations between demographic characteristics, attitude toward general vaccination, risk perceptions, and COVID-19 vaccination intention.

	Crude OR (95%CI)	*p*-Value	Adjusted OR (95%CI)	*p*-Value
**Age groups**				
18–25	-		-	
26–35	0.677 (0.252–1.820)	0.44	0.560 (0.138–2.277)	0.42
36–45	0.515 (0.168–1.576)	0.25	0.373 (0.079–1.753)	0.21
46–55	0.221 (0.047–1.029)	0.054	0.339 (0.043–2.692)	0.31
over 55	0.417 (0.036–4.813)	0.48	0.366 (0.014–9.256)	0.54
**Gender**				
Women	-		-	
Men	0.988 (0.489–1.999)	0.97	2.142 (0.805–5.701)	0.13
**Occupation**				
Physician	-		-	
Other	1.995 (1.129–3.525)	0.02 *	2.099 (0.932–4.724)	0.07
**Attitude toward general vaccination**				
More positive	-		-	
Less positive	6.429 (3.282–12.593)	0.000 ***	5.288 (2.266–12.339)	0.000 ***
**Risk perception**				
Perceived severity of COVID-19 disease				
High	-		-	
Low	2.510 (1.438–4.382)	0.001 **	0.699 (0.313–1.560)	0.38
Perceived susceptibility of COVID-19 disease				
High	-		-	
Low	2.556 (1.457–4.483)	0.001 **	1.450 (0.692–3039)	0.32
Perceived efficacy of COVID-19 vaccine				
High			-	
Low	17.222 (8.538–34.738)	0.000 ***	15.659 (6.798–36.070)	0.000 ***

Significant level: * *p* < 0.05, ** *p* < 0.01, *** *p* < 0.001.

## Data Availability

The data presented in this study are available on request from the corresponding author.

## Supplementary Materials

The following are available online at https://www.mdpi.com/article/10.3390/ijerph19010329/s1, Table S1: summary of the knowledge of recommended occupational vaccine by WHO (2019); Table S2: summary of the knowledge of recommended occupational vaccine by MoH.
