# Supplementary material for: Attitudes towards Mandatory Occupational Vaccination and Intention to Get COVID-19 Vaccine during the First Pandemic Wave among Mongolian Healthcare Workers: A Cross-Sectional Survey
Source: Int J Environ Res Public Health. 2021 Dec 29;19(1):329. doi: 10.3390/ijerph19010329 (PMC8751197; doi:10.3390/ijerph19010329)
Supplement: Supplementary file 1 [file ijerph-19-00329-s001.zip › ijerph-1520161-supplementary.pdf]

Supplementary material

Table S1. summary of the knowledge of recommended occupational vaccine by WHO (2019)

**Variables**	**Total** (N=238)	
	n	%
**Recommended vaccination for HCWs**	(Right answer)	
Diphtheria	75	31.5
Pertussis	89	37.4
Polio	47	19.7
Viral Hepatitis B	211	88.7
Influenza	215	90.3
BCG	92	38.7
Measles	113	47.5
Rubella	84	35.3
Varicella	64	26.9
Meningitis	52	21.8

HCWs- Healthcare workers,
BCG- Bacillus Calmette–Guérin vaccine

Table S2. summary of the knowledge of recommended occupational vaccine by MoH

**Variables**	**Total** (N=238)	
	n	%
**Recommended vaccination for HCWs**	(Right answer)	
Diphtheria	75	31.5
Pertussis	89	37.4
Polio	47	19.7
Viral Hepatitis B	211	88.7
BCG	92	38.7
Influenza	215	90.3
Tetanus	68	28.6

HCWs- Healthcare workers,
BCG- Bacillus Calmette–Guérin vaccine
